# Imaging of Jurassic fossils from the Talbragar Fish Bed using fluorescence, photoluminescence, and elemental and mineralogical mapping

**DOI:** 10.1371/journal.pone.0179029

**Published:** 2017-06-05

**Authors:** Michael Frese, Gerda Gloy, Rolf G. Oberprieler, Damian B. Gore

**Affiliations:** 1 Faculty of Education, Science, Technology and Mathematics, and Institute of Applied Ecology, University of Canberra, Bruce (Canberra), ACT, Australia; 2 Bruker Nano Analytics, Darra (Brisbane), Qld, Australia; 3 CSIRO Australian National Insect Collection, Acton (Canberra), ACT, Australia; 4 Department of Environmental Sciences, Faculty of Science and Engineering, Macquarie University, North Ryde (Sydney), NSW, Australia; University of Michigan, UNITED STATES

## Abstract

The Talbragar Fish Bed is one of Australia’s most important Jurassic deposits for freshwater fishes, land plants and aquatic and terrestrial insects. The site has yielded many well preserved fossils, which has led to the formal description of numerous new species and higher taxa. The excellent preservation of many fossils has allowed detailed anatomical studies, e.g. of the early teleost fish *Cavenderichthys talbragarensis* (Woodward, 1895). Here we report on the fluorescent characteristics and mineral composition of a range of Talbragar fossils. Most specimens fluoresce under ultraviolet, blue and green light. Elemental and mineralogical analyses revealed that the Talbragar fossils consist predominantly of quartz (SiO_2_), a mineral that is likely to account for the observed fluorescence, with trace kaolinite (Al_2_Si_2_O_5_(OH)_4_) in some of the fish fossils. Rock matrices are predominantly composed of quartz and goethite (FeO(OH)). Closer inspection of a plant leaf (*Pentoxylon australicum* White, 1981) establishes fluorescence as a useful tool for the visualisation of anatomical details that are difficult to see under normal light conditions.

## Introduction

The Talbragar Fish Bed near Gulgong in New South Wales (NSW), Australia, is of Upper Jurassic age, ca. 151 Ma, as determined by radiometric dating of zircon crystal inclusions using a sensitive high-resolution ion microprobe (SHRIMP) instrument [1]. This *Lagerstätte* is famous for its fishes and plants, but in recent years it has also yielded fossils of numerous and sometimes beautifully preserved insects.

Since the discovery of the site in 1889 [2], a large number of bony fishes of several species [2, 3] and remains of an elasmobranch shark [4] have been recovered. The most commonly found fish, *Cavenderichthys talbragarensis* (Woodward, 1895) [2], is a primitive teleost [5, 6]. Many fish at the site show signs of tetany (severe postmortem muscular contraction), such as open mouths, erect fins and arched backs, which suggests a lack of oxygen as the cause of death [7, 8]. This and other evidence suggests that the animals died in a single or a series of mass-killing events under hypoxic or anoxic conditions and that they were quickly buried, possibly as a consequence of a large influx of volcanic ash [1, 9]. The absence of common Jurassic marine organisms such as ammonites, belemnites, brachiopods and teeth of marine sharks indicates that the Talbragar Fish Bed was deposited in a freshwater ecosystem.

Another hallmark of the Talbragar *Lagerstätte* is the presence of abundant terrestrial plants [10, 11], suggesting a location near to lush vegetation. The dominant plant fossils are leaves of the conifer *Agathis jurassica* White, 1981, which in recent literature (e.g. [12, 13]) is often placed in the genus *Podozamites*, but this combination is apparently not validly established. The assumption that the Talbragar Fish Bed is a shallow-water deposit close to the shoreline is further supported by the discovery of abundant insect remains (>600 fossils) of both aquatic and terrestrial species from a small area a few hundred metres south of the original excavation site, where most of the larger fish were found (e.g. [14, 15]; older papers are reviewed in [9]). Two spiders have also been found, one of which has been formally described [16]. Taken together, the finds document a complex Jurassic ecosystem, with representatives from a diverse range of fish, insect and plant taxa.

The Talbragar fossils are embedded in ferruginous shale and preserved as compression fossils. A typical fish fossil is a medium-sized (i.e. 40–80 mm long) specimen of *C*. *talbragarensis* with a complete, fully articulated skeleton and most if not all of its scales still in place. The most frequently found insects are one or more as yet undescribed species of aphid-like winged insects belonging to the extinct family Protopsyllidiidae. These small (<8 mm) insects are very delicate but nevertheless almost always completely preserved, with all appendages (i.e. four wings, six legs and two antennae) still attached to the body. Plant fossils are ubiquitous, comprising mainly individual leaves but also, more rarely, small twigs, fruiting bodies or fragments of bark. Most fossils (fish, insects, plants and others) present as “split fossils”, i.e. the rock splits along the fossil (as it represents a natural weakness in the rock), resulting in a “part” and “counterpart” of the specimen. White mineral(s) associated with Talbragar fossils have been described as either kaolinite, (opaline) quartz or a combination of kaolinite and quartz [1, 12]. Some fossils, however, are largely brown, especially larger fish from the northern end of the site. The brown colour in some fossils and the concentric (Liesegang) banding in many Talbragar rocks have been attributed to the deposition of iron minerals [1]. Other fossils are partially or completely black. In these cases, manganese dioxide has been suggested as an infilling mineral, causing a (secondary) blackening of fossils [12]. However, we found it difficult to judge whether or not any of these minerals are indeed involved in the permineralisation of Talbragar fossils, as all statements in the literature so far are either claims unsupported by data (e.g. in [12]) or based on personal communications (e.g. in [1]).

In this paper we use ultraviolet (UV) light-induced fluorescence/photoluminescence, X-ray fluorescence (XRF) and X-ray diffractometry (XRD) to determine the mineral and elemental composition of a range of Talbragar fossils and their rock matrices in greater detail and to assess whether fluorescence can be used to reveal anatomical details in the fossils that are not visible under white light.

## Materials and methods

### Locality and fossils

The Talbragar Fish Bed (32°10’S 149°41’E) is located in the Central Tablelands of New South Wales, approximately 25 km northeast of the town of Gulgong. Though the site is on private land, most of it lies in a reserve administered by the National Parks and Wildlife Services Office in Mudgee, New South Wales. Fossils investigated in this study were either borrowed from the Australian Museum (i.e. the beetle *Rhopalomma stefaniae*, AM F.139992) or collected at the southern end of the site during excavations in 2013–2016. No permits were required to conduct this study, which complied with all relevant regulations. All newly collected specimens were registered with the Australian Museum (AM) in Sydney, New South Wales (numbers AM F.142427 to AM F.142435) and will be stored in the Museum’s collection (Australian Museum, 1 William Street, Sydney, NSW 2010, Australia) after the publication of this work (see S1 Fig for photographs and registration numbers of all new specimens).

### Photoluminescence/Fluorescence

Photoluminescence is the “cold” emission of light in response to the absorption of electromagnetic radiation (UV is commonly used to study photoluminescence but light of different wavelengths can also trigger the phenomenon). Fluorescence is sometimes used in a more restrictive sense to describe luminescence caused by UV light, but the term is used here synonymously with luminescence. Since the 1930s, UV light has been employed to study fossils (reviewed in [17]). In vertebrates, fluorescence has been used to visualise “hidden” bones, suture lines, teeth, feathers and soft tissue (e.g. [18, 19]). Fluorescence has been equally useful to study plant fossils (e.g. [20]) and even to prospect and search for fossils [19, 21, 22]. Tischlinger and Arratia [17] briefly mentioned that Talbragar fish fossils fluoresce under UV light but gave no further details. We investigated the photoluminescence characteristics of a variety of Talbragar fossils (i.e. insects, fish and plants) using UV light and fluorescence microscopy.

Specimens were analysed and photographed under UV light (365 nm) and normal (white) light with a FluorChem 8800 digital (gel) imaging system (Alpha Innotech Corp.). Fossils were further studied with a Nikon Eclipse Ti-U inverted microscope equipped for epifluorescence. Microscope photographs were taken using a Nikon Plan Fluor 4X/0.13 objective and DSFi2 digital camera (for a photograph of the microscope rig, see S2 Fig). To generate and detect fluorescence, the following Semrock filter/mirror combinations were used (S3 Fig): DAPI-1160B (exciter FF01-387/11, emitter FF02 447/50, dichroic FF409-Di03; for the detection of blue fluorescence in UV light), FITC-3540C (exciter FF01-482/35, emitter FF01-536/40, dichroic FF506-Di03; for the detection of green photoluminescence in blue light) and mCherry-B-000 (exciter FF01-562/40, emitter, FF01-641/75, dichroic FF593-Di03; for the detection of red luminescence in green light).

### Standard (light) photography

Photographs were taken with a Canon EOS 5D Mark II camera mounted on a BK Plus imaging system (Visionary Digital^TM^). Images were edited and enhanced in Adobe Photoshop.

### Elemental and mineralogical compositions

Elemental compositions were determined on unprepared specimens using an Olympus Delta Pro XRF spectrometer with a 50-kV Ta anode tube and a 90-s measurement time. The analytical area was a 6 × 4 mm ellipse. Ten elements were reported (Table 1). Repeated measurement of a quartz blank showed all elements except Si to be below detection limit for all measurements. Accuracy was constrained using repeated analysis of two United States National Institute of Standards and Technology certified reference materials, which showed inaccuracies better than 10% for elements more abundant than 0.1 weight (wt) % and better than 20% for trace elements more than double the limit of quantification (LOQ) (Table 1). Compositional data were ratioed to zirconium (Zr) and log-transformed in order to free the data from the constant sum effect and prevent spurious auto-correlations in statistical analysis [23].

**Table 1:** Elemental composition of fossil and matrices of various Talbragar specimens.

SAMPLE TYPE Specimen^*a*^	Elemental composition^*b*^									
	Al %	Si %	P %	S mg/kg	K mg/kg	Ti mg/kg	Cr mg/kg	Mn %	Fe %	Th mg/kg
**ROCK MATRIX**										
Plant1	3.41	26.2	2.29	1026	996	680	82	0.241	59.0	11
Plant2	3.60	27.8	4.04	1159	822	1102	109	0.437	61.5	15
Plant3	2.56	29.9	2.39	633	762	1144	<10	0.181	26.1	10
Plant4	2.79	33.3	1.94	789	737	985	106	0.280	36.7	10
Plant5	2.88	25.3	3.91	810	557	567	71	0.158	28.8	8
Fish1	3.19	31.0	2.24	1254	673	1028	107	0.355	50.5	9
Fish2	3.15	31.1	3.37	794	716	810	76	0.626	43.1	11
Fish3-P	3.25	27.4	2.60	1048	620	779	93	0.612	52.6	13
Fish3-CP	n.r.	n.r.	2.02	n.r.	n.r.	814	113	0.529	55.6	14
**Average**	**3.10**	**29.0**	**2.76**	**939**	**735**	**879**		**0.380**	**46.0**	**11**
**PLANT FOSSIL**										
Plant1	1.91	48.5	2.40	621	185	294	<10	0.178	47.5	12
Plant2	1.09	56.8	3.23	<250	<70	<50	<10	0.515	40.8	10
Plant3	0.25	51.8	1.02	<250	<70	572	25	0.027	7.00	5
Plant4	1.03	51.2	1.83	<250	<70	<50	<10	0.231	22.8	7
Plant5	1.30	42.1	1.43	<250	<70	165	<10	0.191	15.2	1
Plant6-P	n.r.	n.r.	2.28	n.r.	n.r.	389	49	0.581	7.31	11
**Average**	**1.12**	**50.1**	**2.03**					**0.287**	**23.4**	**7.7**
**FISH FOSSIL**										
Fish1	4.02	30.6	2.28	855	572	<50	67	0.393	39.2	12
Fish2	4.20	26.7	2.85	818	315	<50	83	0.610	37.2	13
Fish3-P	5.40	34.1	0.50	651	500	<50	<10	0.332	42.3	13
Fish3-CP	n.r.	n.r.	2.11	n.r.	n.r.	<50	<10	0.315	30.3	11
**Average**	**4.54**	**30.5**	**1.94**	**775**	**462**			**0.413**	**37.3**	**12**
**DARK MATTER**										
Plant3	1.74	39.2	1.24	<250	1462	3183	51	3.41	7.31	6
Plant5	n.r.	n.r.	1.82	n.r.	n.r.	3095	76	4.20	30.3	8
**Average**			**1.53**			**3193**	**64**	**3.81**	**18.8**	**7**

Limits of quantification (LOQ), where relevant, are marked with a “<” sign. Averages are not presented for analytes of which any sample concentrations were <LOQ; n.r. = not reported.

^*a*^ Plant1, *Pentoxylon australicum*; Plant2, 4, 6, *Agathis jurassica*; Plant3, 5, *Rintoulia pinnata*; Fish1–5, *Cavenderichthys talbragarensis*; Plant6-P and Fish3-P are on the same piece of rock; P, part; CP, counterpart. Photographs of all specimens are shown in S1 Fig.

^*b*^ ANOVA (Minitab Express v. 1.5.0) revealed that elemental data were not significantly different among the four types of materials for V (numerical average (av.) = 75 mg/kg), Co (av. = 165 mg/kg), Ni (av. = 65 mg/kg), Cu (av. = 7 mg/kg), Zn (av. = 24 mg/kg), Rb (av. = 9 mg/kg), Sr (av. = 47 mg/kg), Y (av. = 12 mg/kg), Zr (av. = 72 mg/kg), Nb (av. = 10 mg/kg), Mo (av. = 2 mg/kg), Sn (av. = 10 mg/kg), Sb (av. = 6 mg/kg), Ce (av. = 20 mg/kg), Pb (av. = 20 mg/kg) or U (av. = 21 mg/kg) and therefore these elements are not considered further.

Mineralogy was determined using XRD. Diffractograms were collected from 5–90° 2Θ using a PANalytical X'Pert Pro MPD diffractometer, with operating conditions of 45 kV, 40 mA, CuK_α_ radiation, X'Celerator detector, Bragg Brentano geometry and a slew rate of 5° 2Θ per minute. Clasts and their contained fossils were placed on a multi-purpose sample stage, and the reference height was set with a digital micrometer. The stage was fixed and samples were not rotated during data collection, and the irradiated area was set as 5 mm long and 6 mm wide to ensure that measurements of the fossils did not spill over to the adjacent rock matrix (S4 Fig). Diffractograms were collected from the fossil and the adjacent matrix on the clast. Qualitative identification of minerals was undertaken using PANalytical’s Highscore Plus software v2.2.4, with ICDD PDF2 and PAN-ICSD databases. The method detection limit is ~0.1–2 wt %, depending on the sample matrix, mineral crystallinity and instrument settings.

Selected samples were also examined using a Bruker M4 Tornado micro-XRF spectrometer, with mineralogical mapping capability. The M4 Tornado was equipped with a Rh anode tube that was operated at 50 kV and 40 mA. The poly-capillary X-ray optics were able to analyse 25-μm spot sizes. The Advanced Mineral Identification and Characterisation System (AMICS) software (Bruker Corp., Billerica, MA, U.S.A.) compared the X-ray spectra from each pixel with spectra from pure mineral standards and assigned a mineralogical identity to each pixel.

## Results and discussion

### Photoluminescence/Fluorescence

**Fig 1:**
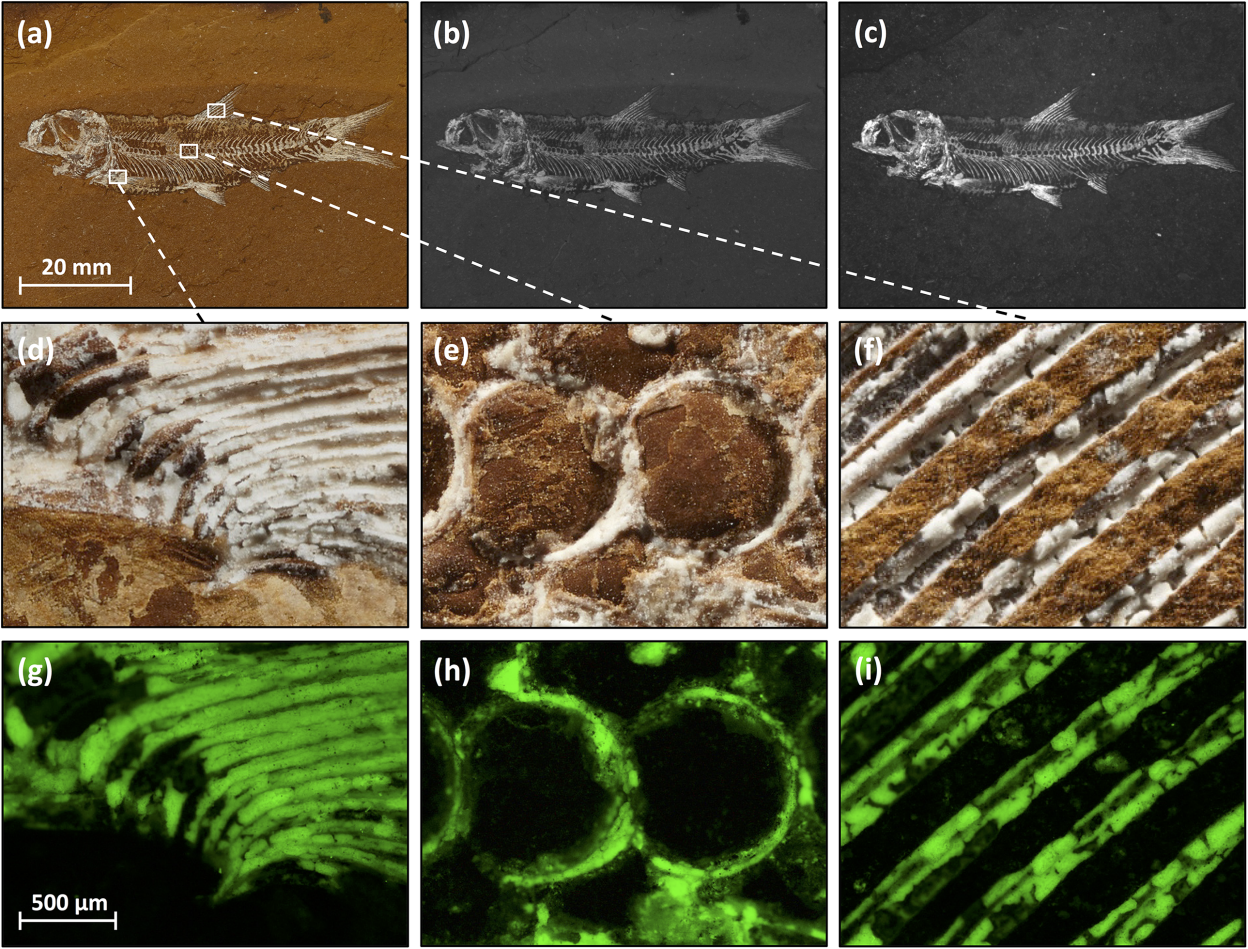
Fluorescence of fish fossils from Talbragar. Teleost fish *Cavenderichthys talbragarensis* (“Fish3”, AM F.142434) photographed under normal (white) light (**a**, **b**, **d**–**f**) or UV light (**c**, **g**–**i**). Photographs were taken with a digital single-lens reflex camera mounted on a motorised stand (**a**, **d**–**f**), a digital camera of a gel-imaging system normally used for analysing ethidium-bromide-stained DNA bands in agarose gels (**b**, **c**) or with a digital camera of a microscope equipped to detect fluorescence/photoluminescence (**g**–**i**). Detail images show pectoral fin bones (**d**, **g**), vertebrae (**e**, **h**) and dorsal fin bones (**f**, **i**). For more images of the same fish, see Figs 7 and 8. Scale bars for panels **a**–**c** and **d**–**i** are given in **a** and **g**, respectively.

UV light generated by a DNA gel document system produced strong fluorescence in a number of Talbragar fish and plant fossils. As an example, we show a skeleton of the fish *Cavenderichthys talbragarensis* (Fig 1A–1C). Illumination with 365-nm UV light triggered strong fluorescence in virtually all fish bones (Fig 1C), which clearly enhanced the contrast between bones and matrix. Also Liesegang bands in the matrix were less visible under UV light. These finding suggest that fluorescence photography can be used to image fossils from the Talbragar Fish Bed.

**Fig 2:**
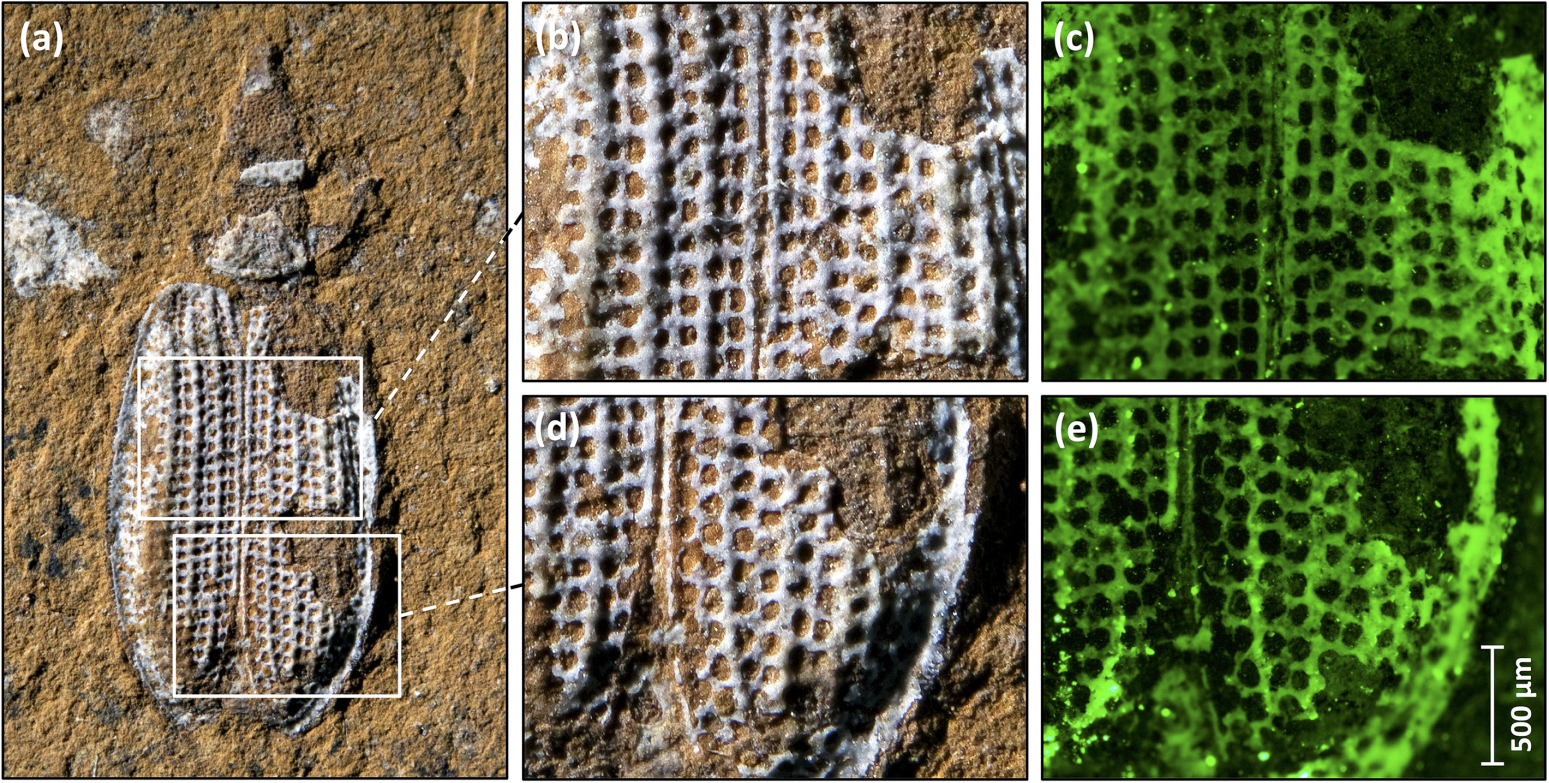
Photoluminescence of an insect fossil. (**a**) The ommatid beetle *Rhopalomma stefaniae* Ashman, Oberprieler & Ślipiński, 2015 (AM F.139992) [14] photographed under normal (white) light, showing a white mineral or a composition of white minerals that partially replaced the organic matter of the head, pronotum and elytra. (**b**, **d**) Higher magnification of the elytra under white light and (**c**, **e**) the same areas exposed to blue light and photographed with a microscope equipped to detect photoluminescence/fluorescence (in this case the emission of green light).

**Fig 3:**
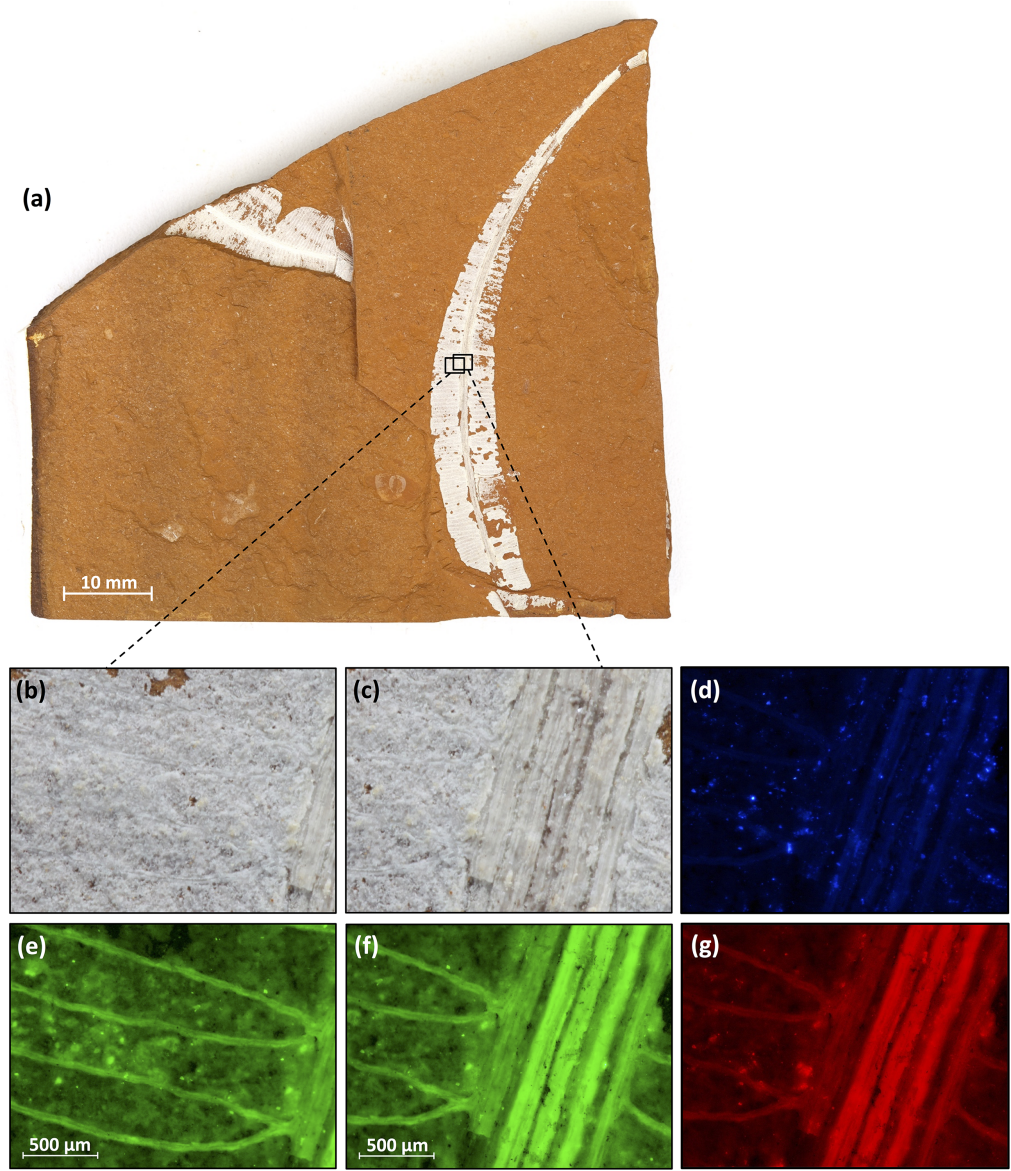
Photoluminescence/Fluorescence of a plant fossil (“Plant1”). (**a**) Rock with two leaves of the gymnosperm *Pentoxylon australicum* (AM F.142427) [11]. (**b**, **c**) Higher magnification of the right leaf, showing lateral and central veins photographed under normal (white) light. (**d**–**g**) Same areas photographed with a microscope equipped to detect photoluminescence/fluorescence: (**d**) fluorescence of violet/blue light after excitation with long-wave UV light; (**e**, **f**) emission of green light after excitation with blue light; and (**g**) emission of red light after excitation with green light. (**e**) Area identical to the part of the leaf shown in (**b**); (**d**, **f**, **g**) area identical to the part of the leaf shown in (**c**). The specimen (i.e. the larger leaf on the right) is identical to “Plant1” in Tables 1 and 2 and Fig 4.

Next we used an epifluorescence microscope to analyse the photoluminescence of the Talbragar fossils. The microscope was a type that is routinely used in molecular biology laboratories for the visualisation of the green fluorescent protein (GFP); immunostained (nonfluorescent) proteins or 4',6-diamidino-2-phenylindole (DAPI)-stained nuclear DNA. Such microscopes can also reveal fine details in a range of Talbragar fossils, including fishes (Fig 1), insects (Fig 2) and plants (Fig 3). The application of fluorescence microscopy was particularly rewarding for the detection of the venation pattern in a leaf specimen of *Pentoxylon australicum* White, 1981. The name of the species *Pentoxylon australicum* is misspelled in the original publication [11], as *P*. *australica*, violating Art. 23.5. of the International Code of Nomenclature for Algae, Fungi and Plants (agreement in gender between adjectival species names and their genus names), because the name *Pentoxylon* is neuter in gender and the adjectival form *australica* is feminine. We therefore here correct the species name to *P*. *australicum*, following Art. 32.2. of the Code. However, we agree with earlier work on the *Pentoxylon* plant that its foliage consisted of elongate leaves with a prominent midrib and numerous lateral veins that may fork near the midrib or more distally [11, 24, 25]. Leaf venation is often of taxonomic value, but in the more mineralised plant specimens from Talbragar the depiction of venation patterns can be difficult to achieve under normal light (Fig 3A–3C). Our findings demonstrate that, in such cases, fluorescent light microscopy can be used to improve the contrast between veins and the surrounding plant tissue (Fig 3D–3G), especially when blue light is used for excitation and green light emissions are imaged (Fig 3E and 3F).

### Elemental and mineralogical compositions

#### Rock matrices

The rock matrices are lower in silicon (Si) and higher in phosphorus (P), sulfur (S), potassium (K), titanium (Ti), chromium (Cr) and iron (Fe) than the plant or fish fossils (Table 1). This is not just a function of the constant sum effect, whereby a decrease in one component (say, Si) causes an increase in the reminder (say, Fe). Instead, ratioing the data (cf. Aitchison 1986 [23]), in this case to Zr, retains these patterns. Rock matrices consist solely of quartz (SiO_2_) and goethite (FeO(OH)); no other trace minerals were found (Table 2) and the minerals hosting elements other than Si and Fe are below the 0.1–2 wt % detection limits of the X-ray diffractometer (Table 1). Diffractogram backgrounds are very low, suggesting that there is little amorphous content in the matrix.

**Table 2:** Mineralogy of fossils and matrices of various Talbragar specimens.

SAMPLE TYPE Specimen^*a*^	Mineral(s)^*b*^
**ROCK MATRIX**	
Plant1	Quartz (SiO_2_), goethite (FeO(OH))
Plant2	Quartz, goethite
Plant3	Quartz, goethite
Plant4	Quartz, goethite
Plant5	Quartz, goethite
Fish1	Quartz, goethite
Fish2	Quartz, goethite
Fish3-P	Quartz, goethite
Fish3-CP	n.r.
**PLANT FOSSIL**	
Plant1	Quartz, goethite
Plant2	Quartz
Plant3	Quartz
Plant4	Quartz
Plant5	Quartz
Plant6-P	n.r.
**FISH FOSSIL**	
Fish1	Quartz, goethite
Fish2	Quartz, kaolinite (Al_2_Si_2_O_5_(OH)_4_), goethite
Fish3-P	Quartz, kaolinite
Fish3-CP	n.r.
**DARK MATTER**	
Plant3	Quartz
Plant5	n.r.

^*a*^ Specimens were the same as in Table 1 and S1 Fig.

^*b*^ Mineral composition based on XRD data.

n.r. = not reported.

#### Plant fossils

**Fig 4:**
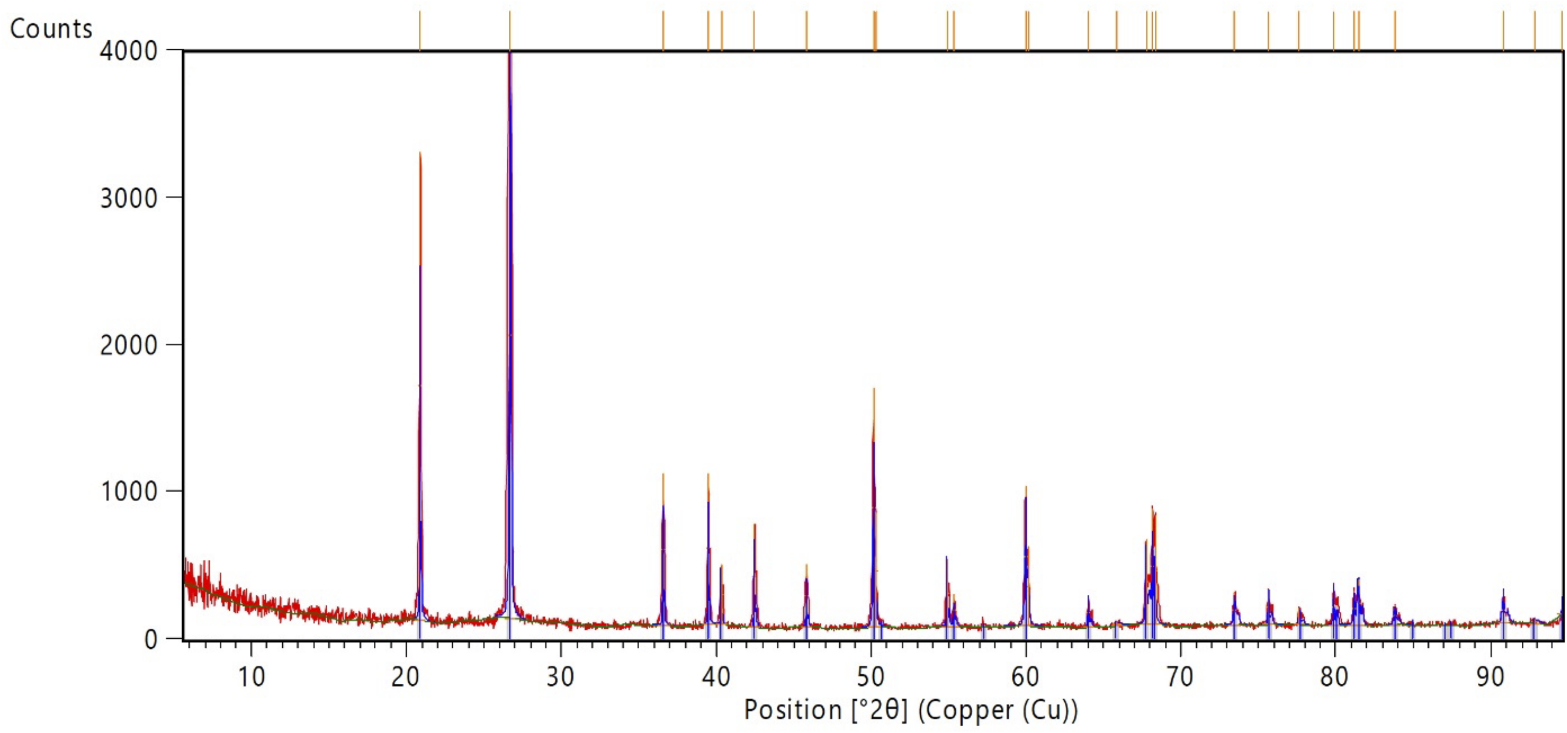
X-ray diffractogram of the plant *Pentoxylon australicum* (“Plant1”) showing quartz. The absence of small unidentified peaks indicates that the fossil has no minor or trace minerals to the limits of detection.

The plant fossils exhibited greater Si and lesser aluminium (Al), P, S, K, Cr, Mn, Fe and Th concentrations compared with the rock matrices and animal fossils (Table 1), commensurate with their composition of quartz (Table 2). One plant fossil appeared to contain a minor quantity of goethite (Table 2), but this was probably due to the thinness of the fossil exposing some of the underlying rock matrix in the measured area. These fossils were exceptional in their purity and monomineralic nature (Fig 4).

#### Fish fossils

The fish fossils contain the greatest concentrations of Al and Mn and the lowest concentrations of Ti, and otherwise were transitional in composition between the rock matrices and the plant fossils (Table 1). This chemistry is consistent with the mineral being quartz, with trace kaolinite (Al_2_Si_2_O_5_(OH)_4_) (Table 2). In some of the X-ray diffractograms, goethite was observed, but again this is probably not in the fossils but in the rock matrix that surrounds the thin fish remains.

#### Dark matter

**Fig 5:**
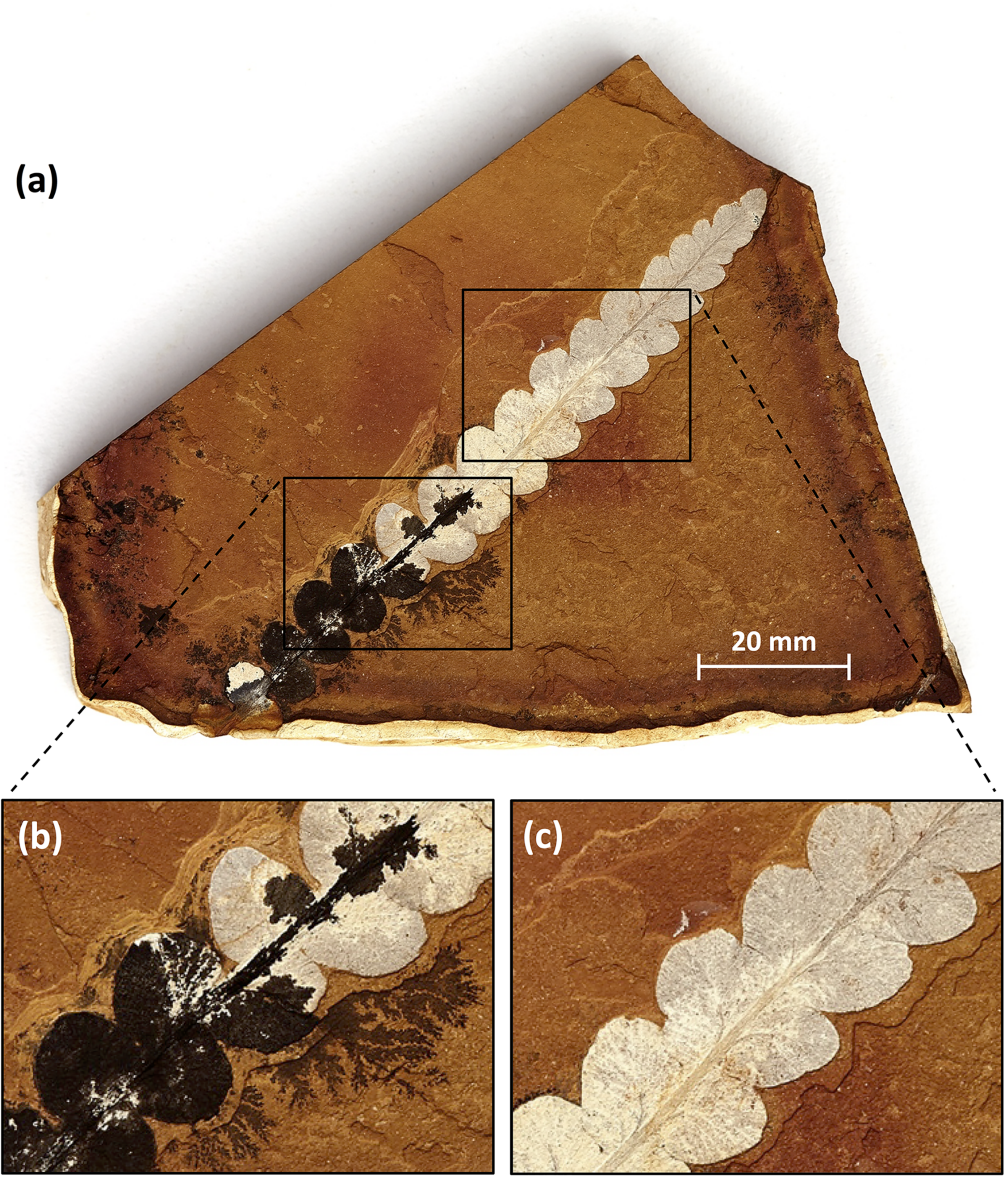
Leaflet of the seed-fern *Rintoulia pinnata* (“Plant3”) showing a colour pattern typical of many fossils of the Talbragar site. (**a**, **b**) Dark manganese dendrites at the edge of the block and around proximal pinnules of the leaf, indicating manganese minerals penetrating the rock along the central vein, causing secondary blackening of proximal pinnules. (**c**) Subtle colour change between median (white) and distal (pale grey) pinnules. The specimen is identical with that named “Plant3” (AM F.142429) in Tables 1 and 2, and Fig 6.

Dark parts of the fossils or matrix of several specimens (Plant2, Plant3, Plant5, Fish3-C) were believed to be manganese-bearing on the basis of a visual inspection; however, manganese-bearing minerals were not detected in the diffractograms and are probably below the XRD detection limits of 0.1–2 wt %. Elemental analyses of the dark matter showed it to be enriched in Mn, to 3–4 wt % versus 0.02–0.6 wt % for the rock matrices and fossils. For example, the dark mineral visible around and on top of the proximal pinnules of the seed-fern *Rintoulia pinnata* (Walkom, 1921) McLoughlin & Nagalingum, 2002 in specimen “Plant3” is almost certainly a thin layer of manganese, forming dendrites into the matrix (Fig 5, Table 1). Manganese infilling of the cavities of the fish fossils, particularly near joints and fractures, has been suggested previously [12] and is consistent with our observations.

#### Elemental and mineralogical mapping

**Fig 6:**
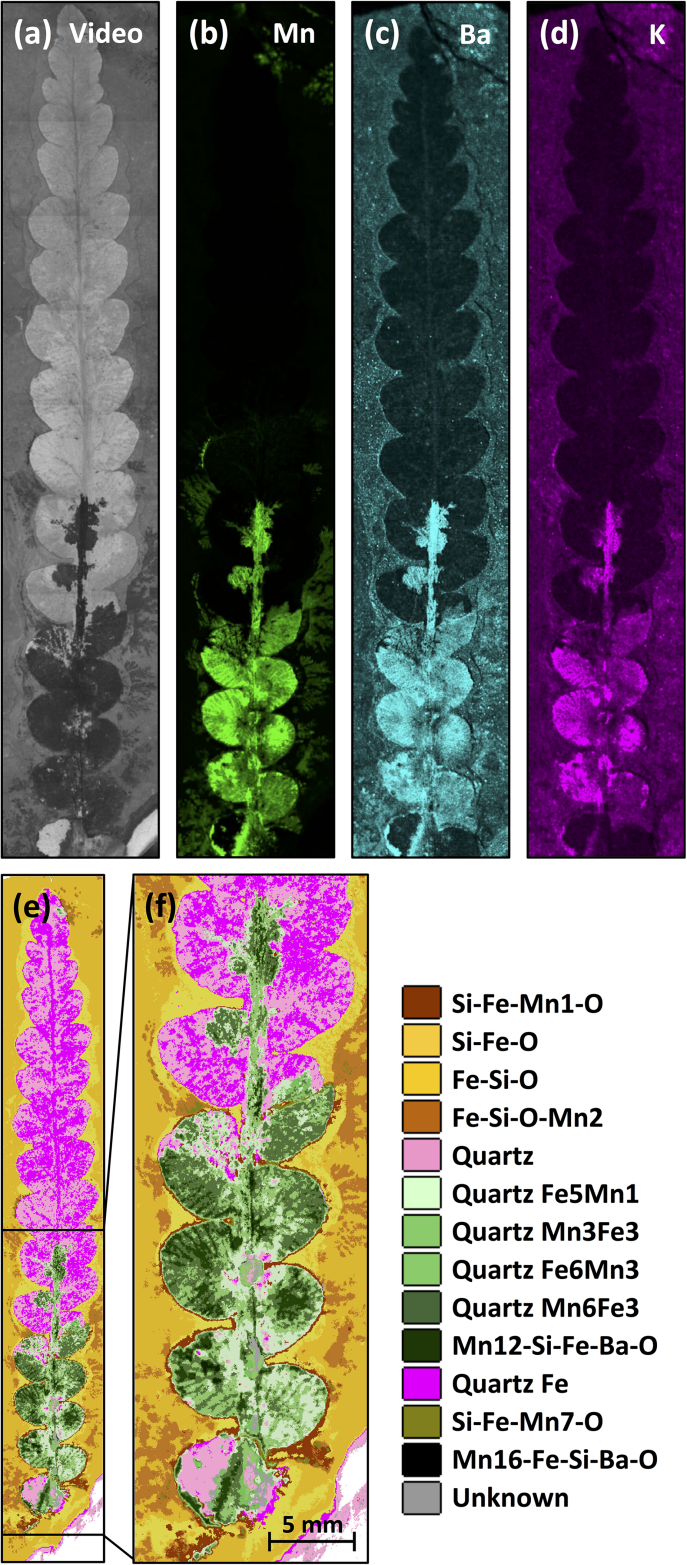
Elemental and mineralogical maps of a *Rintoulia* leaf (“Plant3”). (**a**) Video image of the measured area. (**b**, **c**, **d**) Elemental maps of manganese (Mn), barium (Ba) and potassium (K), respectively. The data show that there is little Mn, Ba or K, except in the dark, manganese-rich area at the leaf base. (**e**, **f**) Mineralogical maps showing the entire leaf and details of the manganese-rich area at the base of the leaf, respectively. The key shows the main mineral of the leaf to be quartz, with intergrown Fe and Mn minerals at the base. The role of Ba and K in these minerals remains unclear.

**Fig 7:**
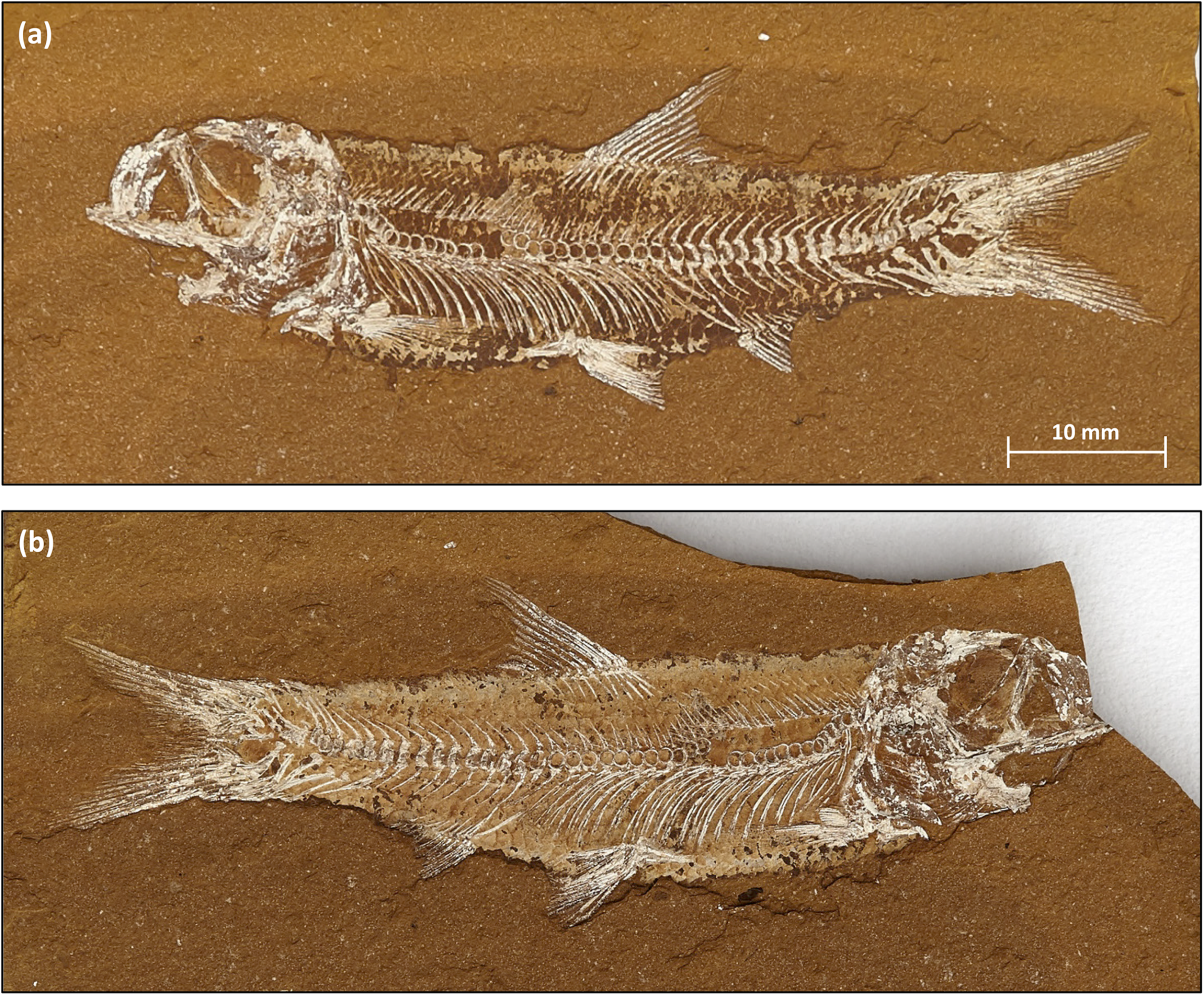
*Cavenderichthys talbragarensis* (“Fish3”). The fish presents as a typical “split fossil” with (**a**) “part” (“Fish3-P”; AM F.142434) and (**b**) “counterpart” (“Fish3-CP”; AM F.142435). In this case, the “part” contains the main body with the majority of the bones exposed, while the “counterpart” contains less bone material but shows more scales. The original fish bones are largely replaced by white minerals. The specimen has not been prepared and is shown as found. The specimens are identical with those named “Fish3-P”and “Fish-CP” in Tables 1 and 2, and Figs 1 and 8.

**Fig 8:**
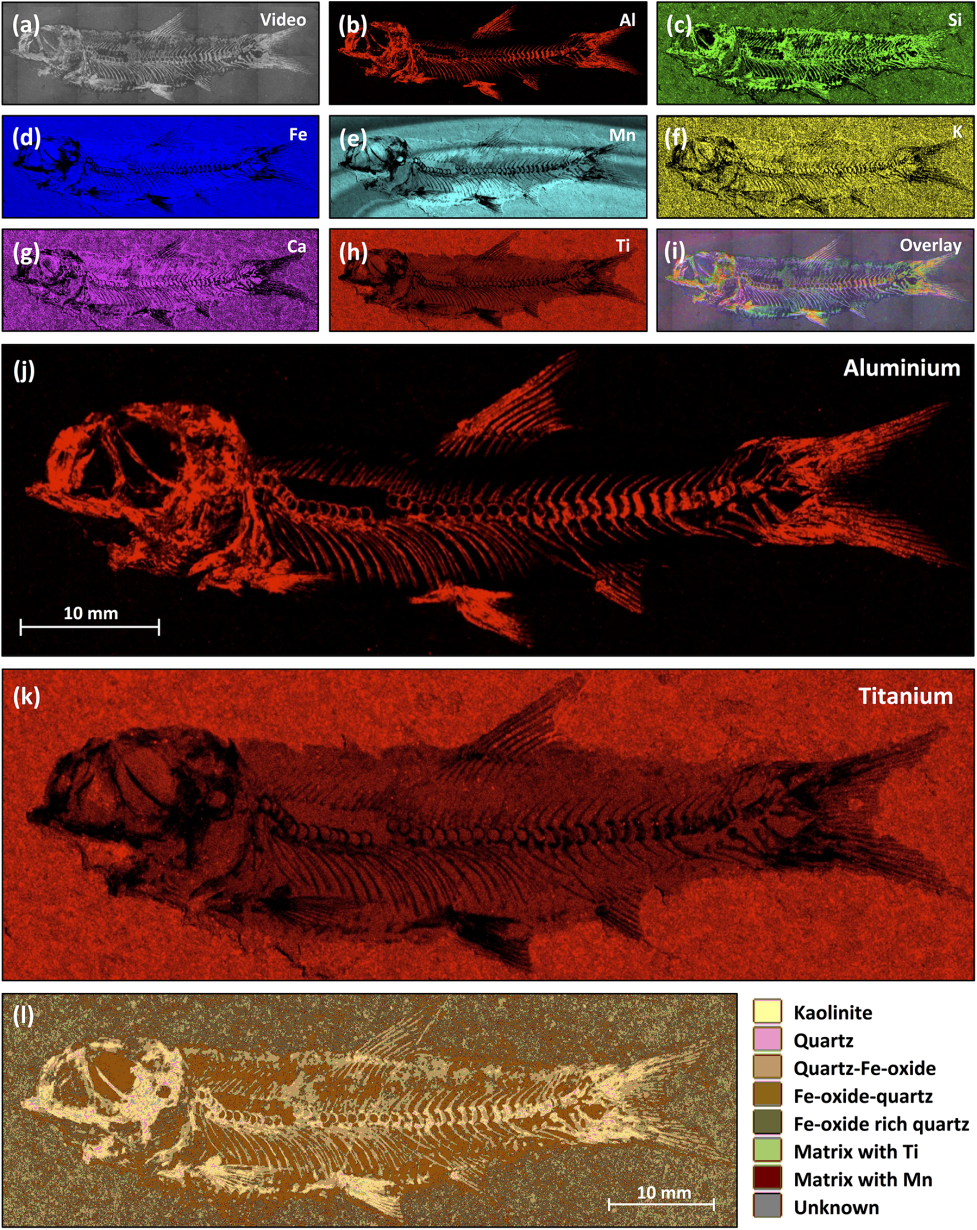
Elemental and mineralogical maps of a *Cavenderichthys* fish (“Fish3-P”). (**a**) Video image of the measured area. (**b**–**h**) Elemental maps for aluminium (Al), silicon (Si), iron (Fe), manganese (Mn), potassium (K), calcium (Ca) and titanium (Ti). (**i**) Video image overlain with elemental maps for Al, Si, Fe, Mn, K, Ca and Ti. The data demonstrate that the fish skeleton consists mainly of Al and Si, whereas Mn shows superb Liesegang banding in an arc through the fossil. (**j**, **k**) Higher magnification of the elemental maps shown in panel **b** and **h**, respectively. (**l**) The mineralogical map shows that the fish fossil consists mainly of kaolinite and quartz, and the matrix consists of quartz with iron oxides, consistent with X-ray diffractograms of the same specimen.

In order to help understand the elemental composition and mineralogy of these specimens better, elemental and mineralogical mapping was undertaken of specimens “Plant3” and “Fish3-P”. Mapping X-ray fluorescence spectrometry of the leaf show K, Mn and Ba outlined in the dark patch at the base of the leaf (Fig 6). The remainder of the leaf is higher in Si than the matrix, lower in Fe and Ba than the matrix and shows no strong contrasts in Al, Mn or K. Mineralogical mapping of these data show pure quartz forming most of the leaf fossil. The darker mineral suspected from visual inspection to be Mn-bearing was identified tentatively Mn minerals in different places (Fig 6). Elemental maps of “Fish3-P” (Figs 7 and 8) show that the fish fossils are rich in Al and Si, elements hosted within kaolinite and quartz respectively. Thus the plant and fish fossils differ elementally and mineralogically, reflecting different diagenetic pathways in the Talbragar Fish Bed, probably as a function of their chemistry during life.

That quartz is the main mineral in plant and fish fossils from Talbragar is consistent with studies from similar oxidising environments (e.g. [26]). Quartz is well-known for its photoluminescence [27], which supports the conclusion that this mineral is largely responsible for the fluorescence of many Talbragar fossils. The diffractograms (e.g. Fig 4) show that the quartz is strongly crystalline and no opal (SiO_2_∙nH_2_O) or other poorly crystalline forms of silicon dioxide are present. Iron oxides in some fossil host rocks are typically associated with hematite (Fe_2_O_3_; [26]), but in strongly oxidising environments iron-bearing oxides may change state from Fe^2+^ to Fe^3+^, producing goethite (FeO(OH)). This is the case with the Talbragar material, and goethite is reported here from the rock matrices from each specimen. This is the first report of goethite from the Talbragar Fish Bed rock matrices.

High-resolution elemental maps generated using XRF spectrometry, such as those in Figs 6 and 8, may be useful to assist in the photographic documentation of fossils. For example, compositional differences in Al between fish bones (high Al concentration) and the matrices of Talbragar rocks (virtually free of Al) produce high-contrast images of the fish skeleton (Fig 8J). Similarly, compositional differences in Ti between the fossils and rock matrices can be exploited to visualise the extent of the space once occupied by the specimens’ soft tissues in the sediment (Fig 8K).

## Conclusions

Fossils of the Talbragar Fish Bed are higher in Si but lower in P, S, K, Ti, Cr, Ba and Fe than surrounding matrices, indicating that silification contributed significantly to the permineralisation of Talbragar fossils. Leaf and fish fossils have similar elevated levels of Si in comparison with the matrix, but only fish bones are rich in Al, a consequence of different diagenetic pathways in plant and fish fossils, probably due to their dissimilar chemical composition in life. The high quartz content of the fossils causes them to fluoresce, a characteristic that can be used for imaging purposes. Also, the sharp contrast between the high concentration of Al in fossil fish bones and the surrounding matrix, which contains virtually no Al but large amounts of the iron oxide goethite, can be used to detect details in fossils that are not otherwise discernible. Detailed knowledge about the elemental composition and permineralisation of fossils thus allows researchers to use a targeted approach for imaging specimens. Our findings establish fluorescence microscopy and elemental mapping by XRF as alternative techniques that can reveal anatomical details of animals and plants fossils from Talbragar in cases where more conventional approaches struggle. We expect that future technological advances may disclose even finer details, and that this can be achieved without time-consuming and potentially destructive preparation. We hope that our work may inspire other researchers to explore new ways to study the unique fossil record of the Talbragar Fish Bed and similar *Lagerstätten*.

## Supporting information

S1 Fig Specimens analysed by XRD. Specimens are identical to those shown in Figs 1, 3 and 5–8, and listed in Tables 1 and 2. (**a**) Rock with two *Pentoxylon australicum* leaves (AM F.142427), “Plant1” is the larger leaf to the right; (**b**) rock (AM F.142428) with *Rintoulia pinnata* leaflet (top, not analysed) and *Agathis jurassica* twig (“Plant2”) underneath (**c**) *R*. *pinnata* leaflet (AM F.142429; “Plant3”); (**d**) *A*. *jurassica* twig (AM F.142430; “Plant4”); (**e**) *R*. *pinnata* leaflet (AM F.142431, “Plant5”); (**f**) rock (AM F.142432) with a leaf (not analysed) and *Cavenderichthys talbragarensis* (“Fish1”); (**g**) *C*. *talbragarensis* (AM F.142433; “Fish2”); (**h**) rock (AM F.142434) with *C*. *talbragarensis* (“Fish3-P”) and *A*. *jurassica* leaves (“Plant6-CP”, not analysed) (**i**) rock (AM F.142435) with *C*. *talbragarensis* (“Fish3-CP”) *A*. *jurassica* leaves (“Plant6-P”). Note that that head of “Fish3-CP” was lost during the course of the studies.
(TIF)

S2 Fig Microscope rig. (**a**) Nikon Eclipse Ti-U inverted (“fluorescence”) microscope and Nikon Plan Fluor DSFi2 digital camera. The monitor shows fluorescence of central and lateral veins of a *Pentoxylon* leaf (“Plant1”). (**b**) Nikon 4X/0.13 objective and the blue excitation light used to generate the image shown in panel **a**.
(TIF)

S3 Fig Semrock filter/mirror combinations used in fluorescence microscopy. Optical characteristics of filters and mirrors that were used to analyse fluorescence in fossils. (**a**) DAPI, (**b**) FITC and (**c**) mCherry filter combinations are normally used to detect the DNA stain 4',6-diamidino-2-phenylindole (DAPI), the fluorescein isothiocyanate (FITC) and the Discosoma fluorophore (mCherry) respectively.
(TIF)

S4 Fig X-ray diffractometry. Sample (“Plant1”) setup in the X-ray diffractometer, showing the multi-purpose sample stage with height set by a digital micrometer. This stage does not rotate the sample. Programmable slits were used to constrain the irradiated area to 5 mm long and 6 mm wide.
(TIF)
