# Supplementary figures and images for: Imaging of Jurassic fossils from the Talbragar Fish Bed using fluorescence, photoluminescence, and elemental and mineralogical mapping
Source: PLoS One. 2017 Jun 5;12(6):e0179029. doi: 10.1371/journal.pone.0179029 (PMC5459505; doi:10.1371/journal.pone.0179029)

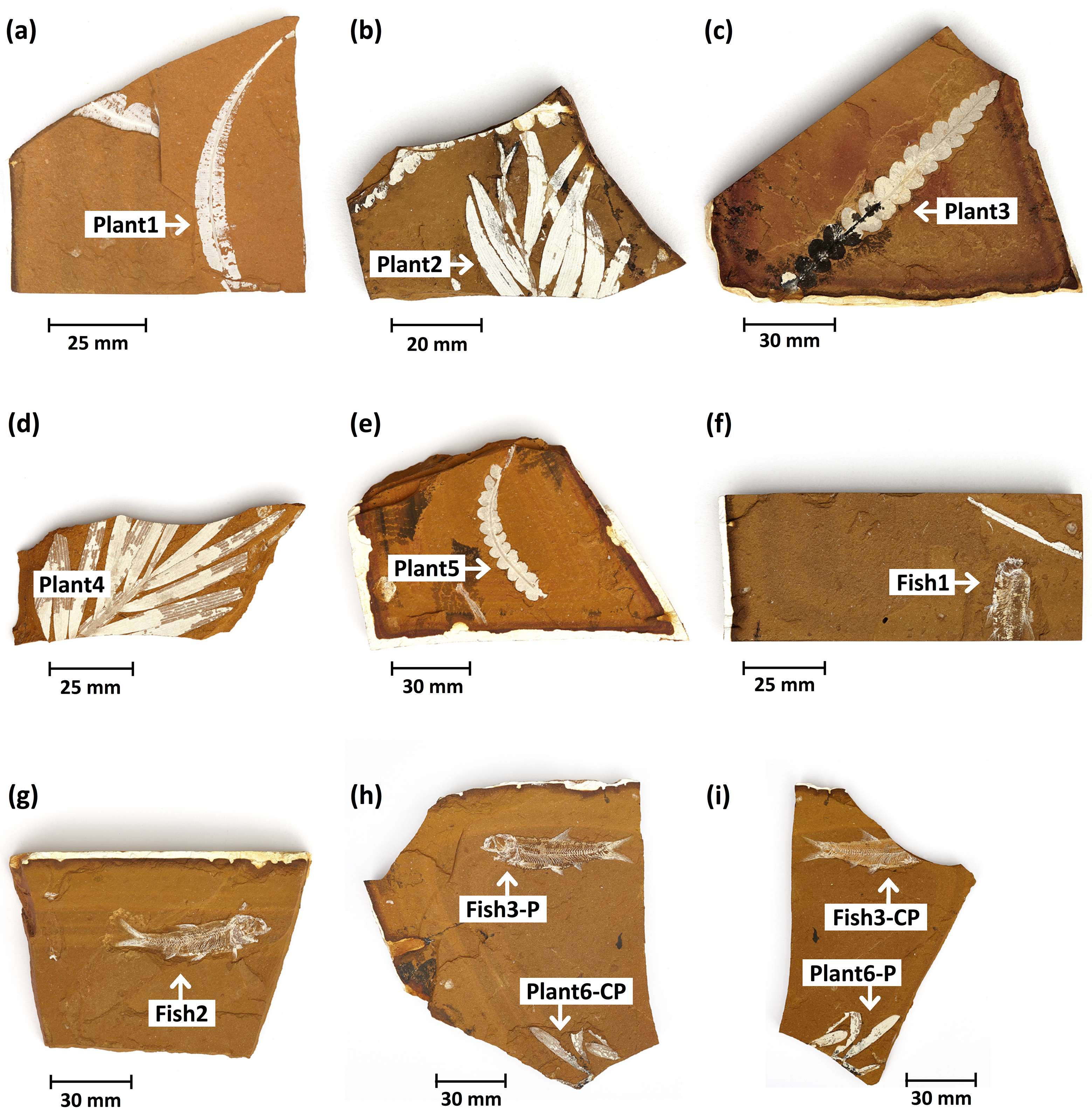

Supplement: S1 Fig — Specimens are identical to those shown in Figs 1, 3 and 5–8, and listed in Tables 1 and 2. (a) Rock with two Pentoxylon australicum leaves (AM F.142427), “Plant1” is the larger leaf to the right; (b) rock (AM F.142428) with Rintoulia pinnata leaflet (top, not analysed) and Agathis jurassica twig (“Plant2”) underneath (c) R. pinnata leaflet (AM F.142429; “Plant3”); (d) A. jurassica twig (AM F.142430; “Plant4”); (e) R. pinnata leaflet (AM F.142431, “Plant5”); (f) rock (AM F.142432) with a leaf (not analysed) and Cavenderichthys talbragarensis (“Fish1”); (g) C. talbragarensis (AM F.142433; “Fish2”); (h) rock (AM F.142434) with C. talbragarensis (“Fish3-P”) and A. jurassica leaves (“Plant6-CP”, not analysed) (i) rock (AM F.142435) with C. talbragarensis (“Fish3-CP”) A. jurassica leaves (“Plant6-P”). Note that that head of “Fish3-CP” was lost during the course of the studies. (TIF) [file pone.0179029.s001.tif]

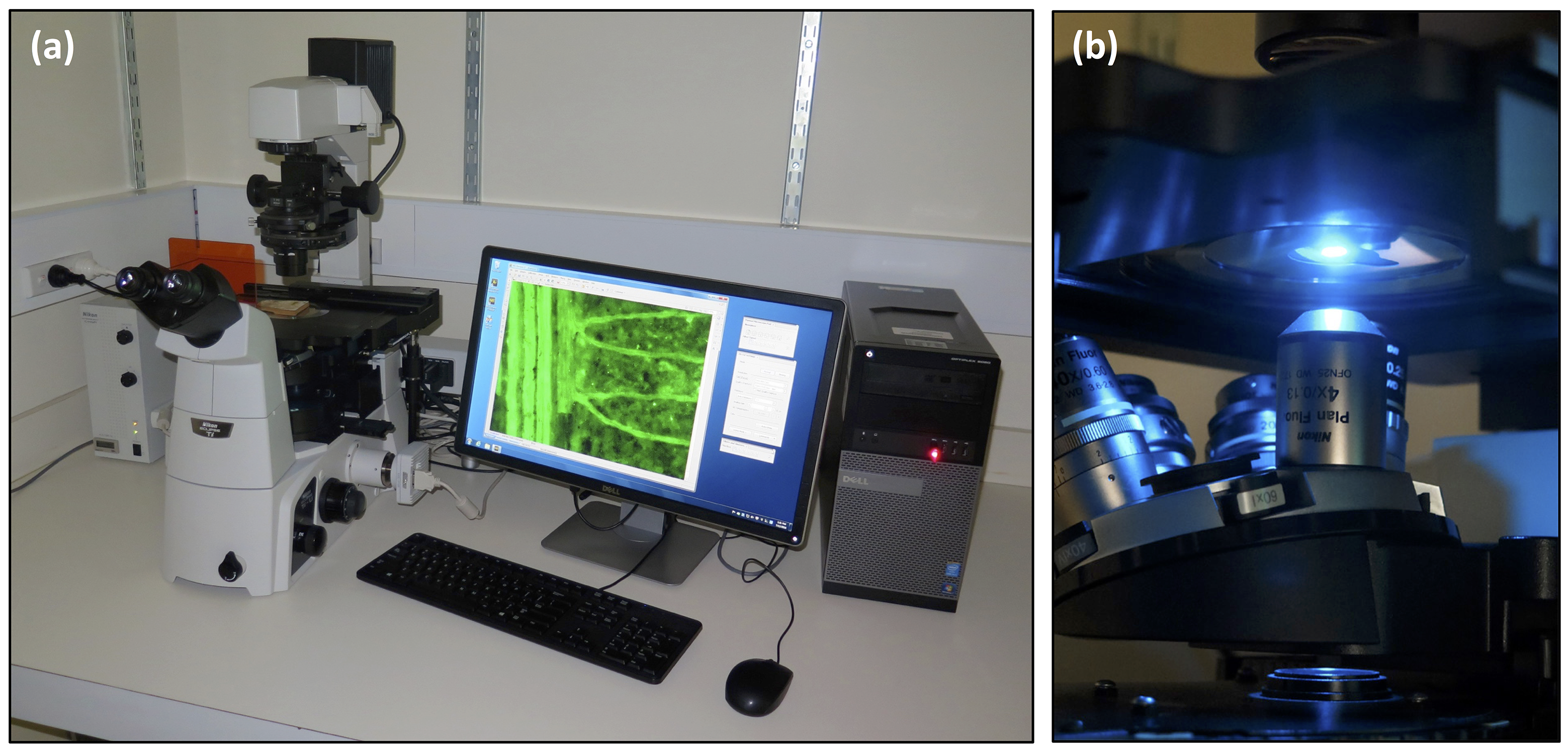

Supplement: S2 Fig — (a) Nikon Eclipse Ti-U inverted (“fluorescence”) microscope and Nikon Plan Fluor DSFi2 digital camera. The monitor shows fluorescence of central and lateral veins of a Pentoxylon leaf (“Plant1”). (b) Nikon 4X/0.13 objective and the blue excitation light used to generate the image shown in panel a. (TIF) [file pone.0179029.s002.tif]

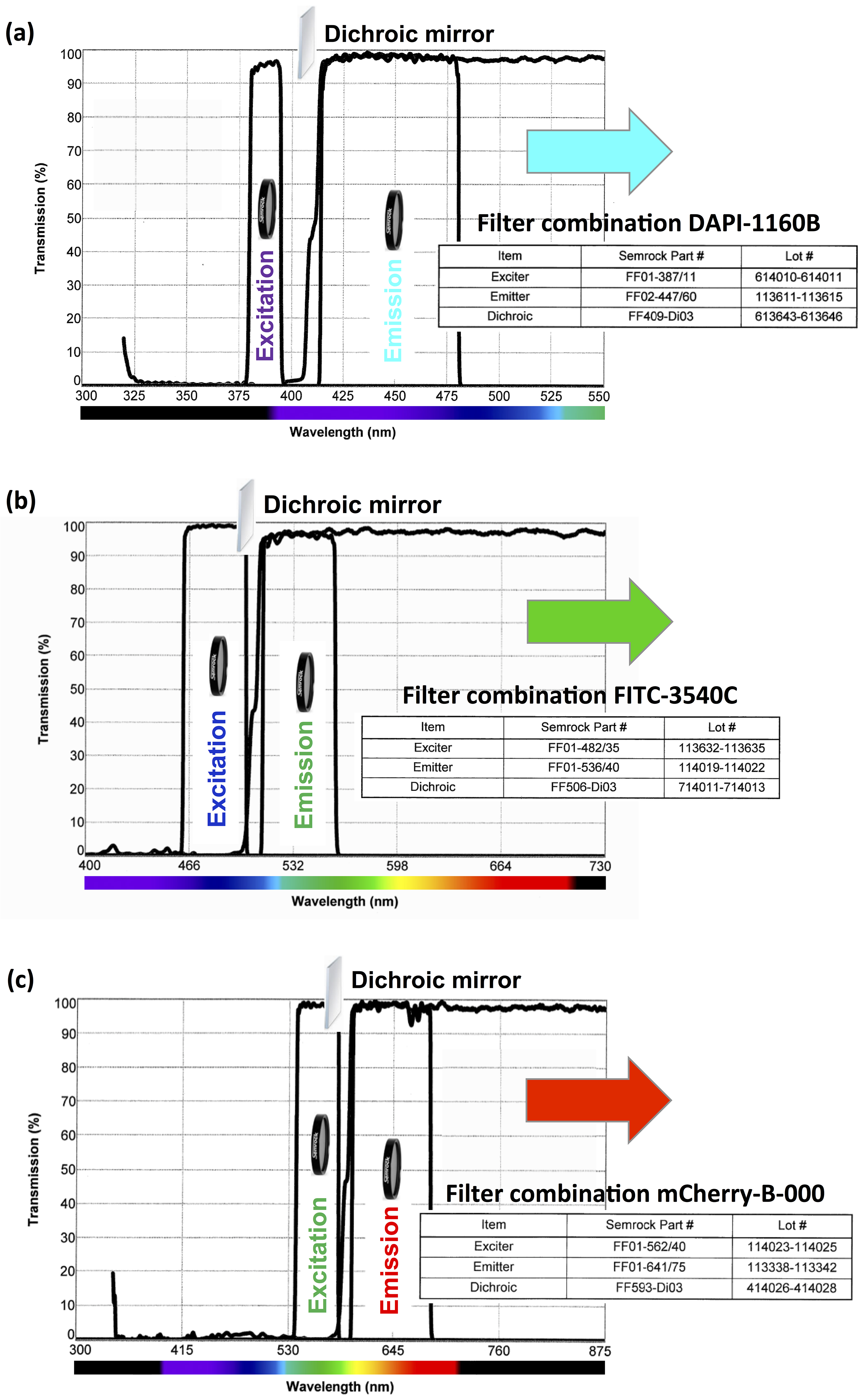

Supplement: S3 Fig — Optical characteristics of filters and mirrors that were used to analyse fluorescence in fossils. (a) DAPI, (b) FITC and (c) mCherry filter combinations are normally used to detect the DNA stain 4',6-diamidino-2-phenylindole (DAPI), the fluorescein isothiocyanate (FITC) and the Discosoma fluorophore (mCherry) respectively. (TIF) [file pone.0179029.s003.tif]

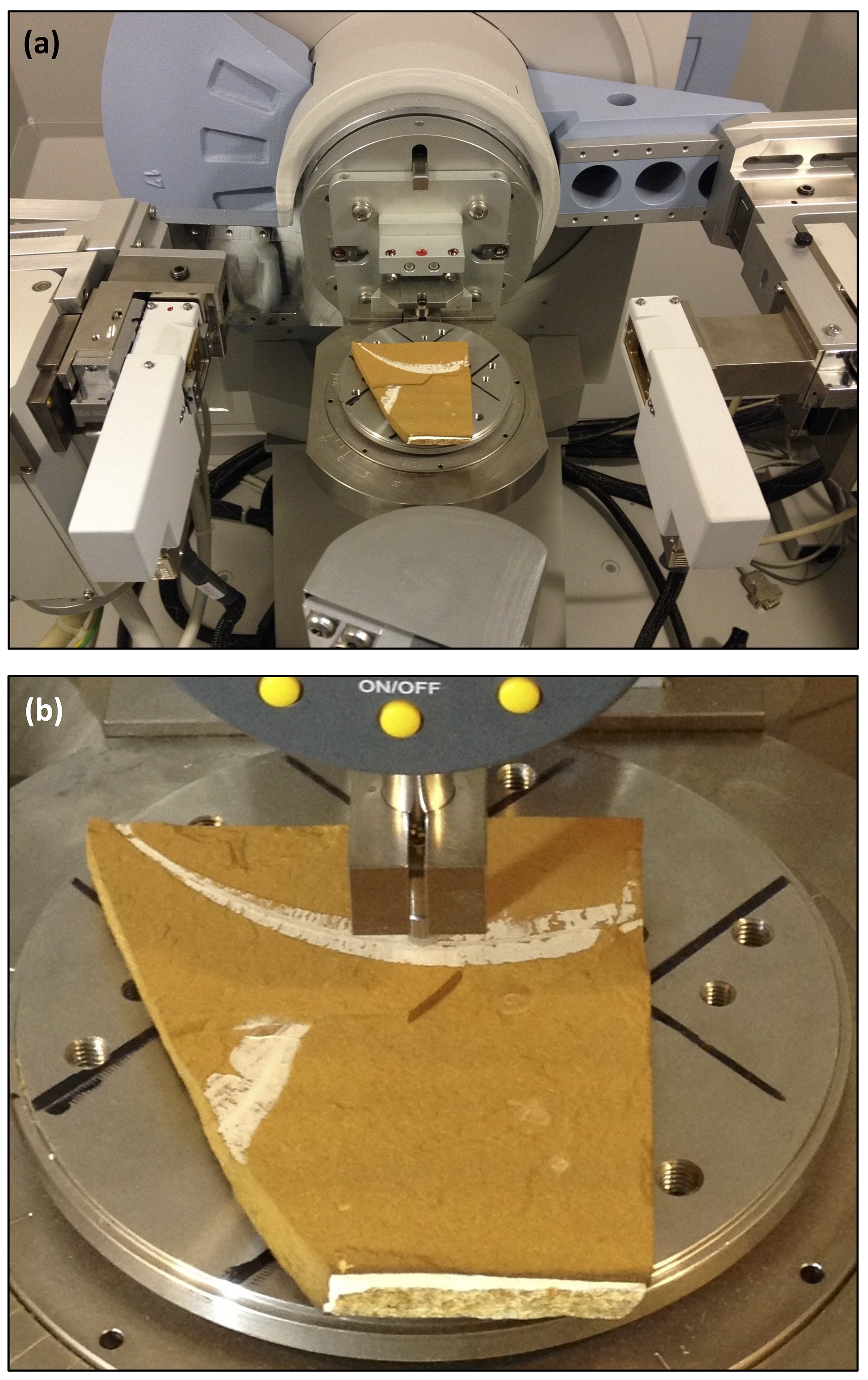

Supplement: S4 Fig — Sample (“Plant1”) setup in the X-ray diffractometer, showing the multi-purpose sample stage with height set by a digital micrometer. This stage does not rotate the sample. Programmable slits were used to constrain the irradiated area to 5 mm long and 6 mm wide. (TIF) [file pone.0179029.s004.tif]
